# Single-cell sequencing reveals a senescent immune landscape in bone marrow lesions inducing articular cartilage damage in osteoarthritis

**DOI:** 10.1038/s41413-025-00467-4

**Published:** 2025-11-21

**Authors:** Pengqiang Lou, Xiaoyan Lu, Mengyin Li, Yue Yao, Xin Shao, Dan Shou, Xiaohui Fan, Peijian Tong, Yang Zhang

**Affiliations:** 1 Institute of Orthopaedics and Traumatology, The First Affiliated Hospital of Zhejiang Chinese Medical University (Zhejiang Provincial Hospital of Chinese Medicine), Hangzhou, 310053 China; 2 Pharmaceutical Informatics Institute, College of Pharmaceutical Sciences, Zhejiang University, Hangzhou, 310058 China; 3 State Key Laboratory of Chinese Medicine Modernization, Innovation Center of Yangtze River Delta, Zhejiang University, Jiaxing, 314102 China; 4 School of Pharmaceutical Sciences, Zhejiang Chinese Medical University, Hangzhou, 310053 China

**Keywords:** Bone, Pathogenesis

## Abstract

Bone marrow lesions (BML) are early signs of osteoarthritis (OA) and are strongly correlated with the deterioration of cartilage lesions. Single-cell RNA sequencing (scRNA-seq) analyses were performed on BM from non-BML and BML areas and articular cartilage from intact and damaged areas to explore BML landscape and BML-cartilage crosstalk. We revealed the immune landscape of BM in non-BML and BML, and the transition to pro-inflammatory states of clusters in BMLs, such as classical monocytes and non-classical monocytes. Non-classical monocytes have high inflammation, OA gene signatures, and senescence scores, and are potential primary clusters promoting OA progression. Histological signs of OA related to the cellular landscape in damaged cartilage were identified, including PreFC exhaustion. The BM-cartilage crosstalk at the cell-cell interaction (CCIs) level and the TNF signal transmitted by non-classical monocytes are the critical CCIs in BML-induced cartilage damage, and PreFC is one of the primary receivers of the signal. We further validated the higher senescence level of non-classical monocyte and FC-2 in OA mice, compared with classical monocyte and PreFC, respectively. Transcription factor 7 like 2 (TCF7L2) was identified as a shared transcription factor in the senescence of monocytes and chondrocytes, facilitating the development of the senescence-associated secretory phenotype (SASP). Therefore, senescent non-classical monocytes promote BMLs and inflammation and senescence of chondrocytes by modulating BML–cartilage crosstalk in OA, with TCF7L2 serving as a regulator.

## Introduction

Osteoarthritis (OA) is an age-related degenerative disorder affecting knee joints and influences all knee tissues, such as cartilage, meniscus, synovium, subchondral bone, and bone marrow.^1^ Pathologically, OA progression involves bone marrow lesions (BML) development, abrasion of cartilage and meniscus, inflammation microenvironment changes in the synovium and synovial fluid, and gradual remodeling of subchondral bone. Notably, BMLs are early signs of OA,^2^ indicators of OA onset, and early treatment targets.^3^ A strong correlation exists between the enlargement of the subchondral high-signal region on magnetic resonance imaging (MRI), indicative of BML, and a decrease in cartilage volume within the corresponding BML-affected area.^4^ Furthermore, the expansion of BML is accompanied by the spatial deterioration of cartilage,^5–7^ indicating a potential relationship between BML and cartilage erosion.^8^ Meanwhile, knees with BML progression were associated with an increased risk of OA compared with BML-free knees and knees with BML regression.^9^

However, most current research on OA focuses on the cartilage and synovium, with a lesser focus on the role of BML in OA progression. Direct molecular communication between cartilage and subchondral bone has been demonstrated and elevated in OA progression.^10^ Evidence indicates small-molecule communication through cortical endplates and calcified cartilage.^10^ Vascular channels establish a direct connection between the bone marrow (BM) and joint space in proximity to the distal end of the knee ligament. These channels facilitate the transmission of signals and cellular infiltration among the BM, joint fluid, chondrocytes on the joint surface, and synovial membrane.^11^ Simultaneously, the immune cell composition of the knee joint and synovium is influenced by immune cells recruited from the BM.^12^ Therefore, alterations in the BML microenvironment may contribute to OA cartilage degeneration through cartilage–subchondral bone crosstalk.

Nevertheless, there is a lack of understanding of the pathogenic processes shared by BMLs and cartilage. Prominent immune cell infiltration is an important pathological feature of BMLs,^13^ and inflammation is critical in OA development. T cells, B cells, monocytes, dendritic cells, neutrophils, and macrophages, have been proposed to contribute to OA pathogenesis,^11,14–18^ suggesting a role of the immune landscape in BMLs and OA progression. Therefore, given the presence of direct molecular signaling from the BM in cartilage and subchondral bone, exploring mechanisms of BML-cartilage crosstalk may provide a rationale for future intra-articular or intraosseous therapies. Bioinformatic analyses of knee OA, including bulk RNA sequencing, have revealed dysregulation of the biological process in knee OA.^19–21^ Bulk RNA sequencing averages global gene expression changes between tissues that are often highly heterogeneous in cell type composition, but ignores the contribution of cells in different OA states to disease development and their respective roles. Single-cell RNA sequencing (scRNA-seq) addresses these limitations.^22^ Several studies have used scRNA-seq to study OA development,^23^ including analyses of the heterogeneity of cell populations in the meniscus^24^ and synovium.^25^ However, few scRNA-seq studies have focused on the effect of BMLs and their immune landscape on the cartilage in OA.^26–29^ Hence, we performed scRNA-seq analysis of cartilage and BM samples in OA to determine the BML–cartilage crosstalk and to investigate separate and shared mechanisms in cellular homeostasis and OA pathogenesis.

## Result

### The immune cellular landscape in non-BML and BML regions

**Fig. 1:**
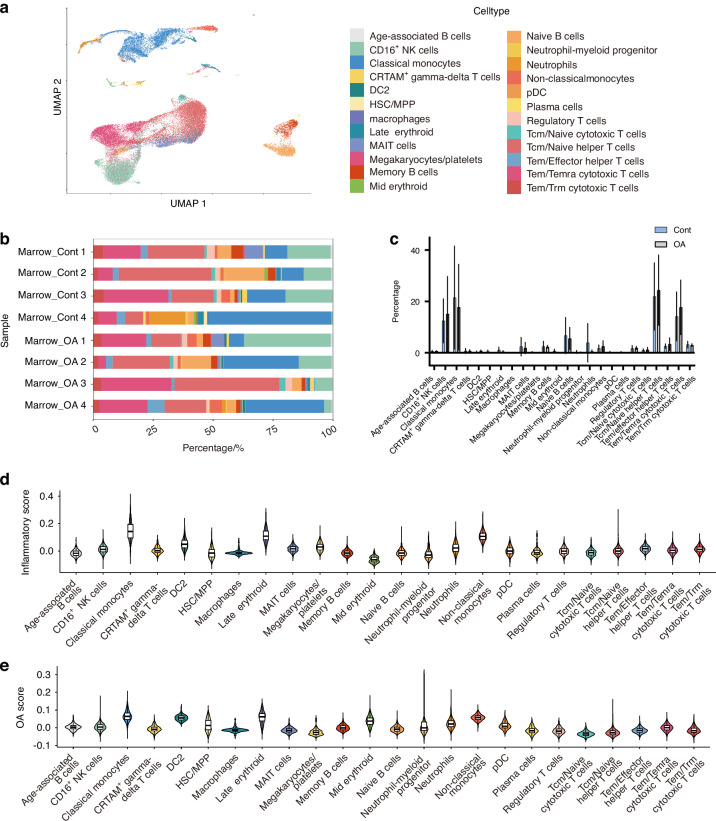
Single-cell sequencing of BM from non-BML and BML areas. **a** Visualization of clustering by Uniform Manifold Approximation and Projection (UMAP) plot of integrated non-BML (*n* = 4, CONT) and BML (*n* = 4, OA) BM samples. **b** The quantification of individual donor contribution to each cluster is shown in the cell count percentage of each sample. **c** Statistical analysis of the proportion of each cluster in the various samples in the CONT and OA groups. ns *P* > 0.05 by Wilcoxon test. **d** An inflammation gene signature score was assigned to each cell in the integrated dataset, and the scores were visualized on a violin plot split by cluster. **e** An OA gene signature score was assigned to each cell in the integrated dataset, and the scores were visualized on a violin plot split by cluster. Tem effector memory T cells, Trm resident memory T cells, Temra effector memory re-expressing CD45RA T cells, Tcm central memory, MAIT mucosal-associated invariant T, pDC pre-dendritic cells, HSC/MPP hematopoietic stem cell and multipotent progenitor, DC2 conventional type 2 dendritic cells, NK Natural killer

We performed scRNA-seq on BM samples from the non-BML and BML areas obtained from four donors who underwent unicompartmental knee replacement. We obtained 43 432 cells for the analysis (Fig. S1), including 23 657 cells from non-BMLs, and 19 775 cells from BMLs. Celltypist^30^ was used to annotate cell clusters automatically, and we referred to previously published studies to annotate each cell cluster^31–34^ (Fig. S2, Table S1). We identified 24 cell clusters, including six myeloid cell clusters: pre-dendritic cells (pDC), conventional type 2 dendritic cells (DC2), classical monocytes, non-classical monocytes, neutrophils, neutrophil–myeloid progenitors; eight T-cell clusters: effector memory/resident memory (Tem/Trm) cytotoxic T cells, effector memory/effector memory re-expressing CD45RA (Tem/Temra) cytotoxic T cells, effector memory/effector (Tem/Effector) helper T cells, central memory/naive (Tcm/Naive) helper T cells, central memory/naive (Tcm/Naïve) cytotoxic T cells, regulatory T cells, mucosal-associated invariant T (MAIT) cells, CRTAM^+^ gamma-delta T cells; four B-cell clusters: naive B cells, memory B cells, age-associated B cells, Plasma cells; two erythroid cell clusters: mid erythroid, late erythroid; hematopoietic stem cell and multipotent progenitor (HSC/MPP); megakaryocytes/platelets; CD16^+^ Natural killer (NK) cells; and a small quantity of macrophages clusters (Fig. 1a, b). Cluster identification revealed a decrease in the proportion of classical monocytes and an increase in the proportion of non-classical monocytes, CD16^+^ NK cells, Tcm/naïve helper T cells, and Tem/Temra cytotoxic T cells (Table S2). However, there was no statistically significant change in the proportions of these clusters between non-BMLs and BMLs. (Fig. 1c).

To illustrate the contribution of cell clusters in OA, we used the inflammation gene set and OA-association gene set^24^ (Table S3) to assess the pro-inflammation and the contribution to OA progression of the BM clusters. We found that macrophage, non-classical monocytes, and classical monocytes have a high score in inflammatory (Fig. 1d) and OA gene signature (Fig. 1e). Meanwhile, non-classical monocytes and classical monocytes have higher inflammation and OA scores in the BMLs (OA) group compared with the non-BMLs (CONT) group (Fig. S3a, b). However, the macrophage cluster has a low proportion in samples (<0.1%) and is insufficient to characterize its role in the progression of BMLs. Furthermore, the enrichment results of clusters’ upregulated genes in BMLs reveal that the inflammation pathway is active in classical monocytes, specifically the IL-17 signaling pathway, and TNF signaling pathway. Interestingly, in BMLs, classical monocyte shows cellular senescence pathway up-regulated (Fig. S4a). Non-classical monocytes also show the up-regulated inflammatory pathway (Fig. S4b). Therefore, we considered that the classical monocytes and non-classical monocytes are the most pro-inflammation clusters and the main clusters contribute to BMLs and OA progression.

We analyzed the cellular communication patterns within the BM and enriched 25 cell-cell interactions (CCIs) (Fig. S5a). CD16^+^ NK cells, classical monocytes, Tcm/Naïve helper T cells, and Tem/Temra cytotoxic cells were the major signal senders in the BM tissues, and classical monocytes were the predominant signal receivers in the BM cells. Immune-related signals were the main category across all CCIs, including ANNEXIN,^35^ MIF,^36^ GALECTIN,^37^ and CCL.^38^

### The cellular landscape in articular cartilage

**Fig. 2:**
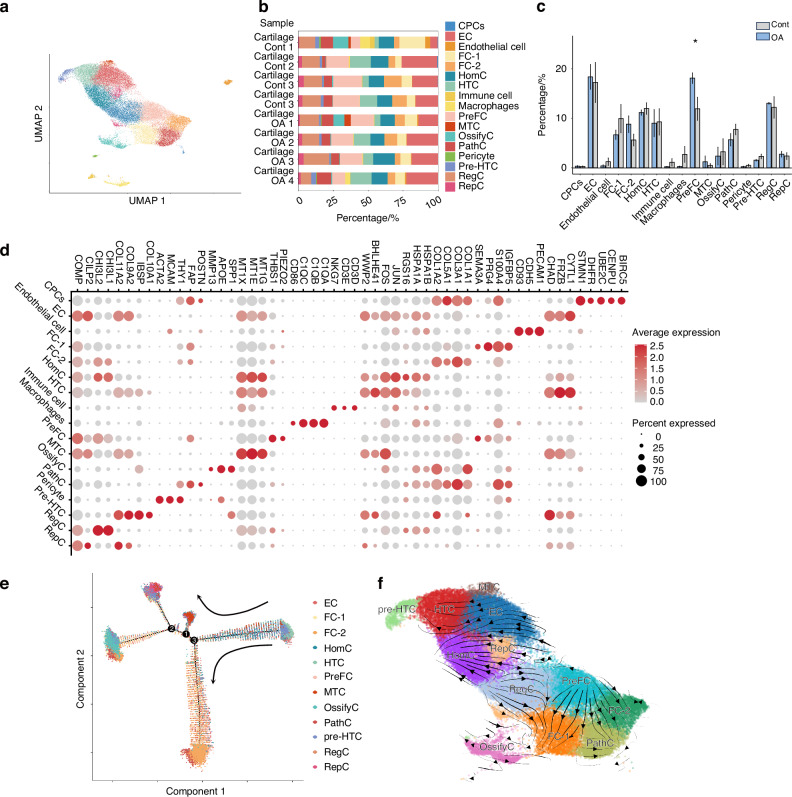
Single-cell sequencing of intact and damaged human articular cartilage. **a** Visualization of clustering by Uniform Manifold Approximation and Projection (UMAP) plot of integrated intact (*n* = 4, CONT) and damaged (*n* = 4, OA) articular cartilage. **b** The quantification of individual donor contribution to each cluster is shown in each sample’s cell count percentage. **c** Statistical analysis of the proportion of each cluster in the various samples in the CONT and OA groups. * *P* < 0.05 by Wilcoxon test. **d** The dot plot shows the mean expressions of the marker genes for cell subsets. **e** Cell distribution of chondrocytes along the pseudo-time trajectory. **f** The RNA velocity of the chondrocytes RegC regulatory chondrocytes, EC effector chondrocytes, FC fibrocartilage chondrocytes, pathC pathogenic chondrocytes, preHTC prehypertrophic chondrocytes, HTC hypertrophic chondrocytes, HomC homeostatic chondrocytes, MTC metallothionein chondrocytes, OssifyC ossify chondrocyte, PreFC THBS1^+^ chondrocyte, CPC cartilage progenitor cells. SZ superficial zone, MZ middle zone, DZ deep zone. *P < 0.05, **P < 0.01, ****P < 0.000 1 by unpaired Student’s *t*-test

We collected intact (*n* = 4) and damaged (*n* = 4) cartilage samples from four donors. Overall, 54 412 cells were analyzed, including 23 617 cells from intact cartilage, and 30 796 cells from damaged cartilage (Fig. S6), and 16 clusters were identified (Fig. 2a, b). We identified previously described chondrocyte populations,^24,39–44^ including regulatory chondrocyte (RegC), effector chondrocyte (EC), fibrocartilage-1 (FC-1), fibrocartilage-2 (FC-2), pathogenic chondrocyte (pathC), prehypertrophic chondrocyte (preHTC), hypertrophic chondrocyte (HTC), homeostatic chondrocyte (HomC), metallothionein chondrocyte (MTC), reparative chondrocyte (RepC), cartilage progenitor cells (CPC), ossify chondrocyte (OssifyC) and prefibro chondrocyte (PreFC). And we noticed that PreFC highly expresses THBS1 and PIEZO2, and can be identified as the marker of PreFC. OssifyC highly expresses SPP1, APOE, and MMP13 (Fig. S7a, c). We also identified non-chondrocyte cell populations, including macrophages, endothelial cells, pericytes, and other immune cells in all donors’ samples. The markers used to identify the cell subsets are shown as a dot plot (Fig. 2d) and listed in Table S4. Cluster identification revealed a significant decrease of PreFC in damaged cartilage. Meanwhile, there is a decrease of EC, MTC, and RegC in damaged cartilage, as well as an increase in FC-1, pre-HTC, PathC, OssifyC, endothelial cells, immune cells, and macrophage subsets, although there was no statistically significant change in the proportions of these clusters (Fig. 2c, Table S5).

Recent studies have found that PreFC prevents cartilage degeneration.^44^ The further enrichment results show that PreFC is involved in a variety of biological processes including, extracellular matrix organization, cartilage development, positive regulation of chondrocyte differentiation, chondrocyte differentiation, collagen fibril organization, and the positive regulation of mesenchymal stem cell differentiation, are vital in the cartilage homeostasis. Meanwhile, the PreFCs also participate in OA-related biological processes, such as angiogenesis, ossification, positive regulation of neuron differentiation, positive regulation of osteoblast differentiation, and detection of mechanical stimuli (Fig. S7b). Furthermore, we performed GO biological process enrichment analysis of up-regulated genes of PreFC in damaged cartilage. The up-regulated biological process includes the response to various stimuli, mitochondria-related biological processes, and the regulation of the OA process (Fig. S8a, b) The biological processes of OssifyC are involved in endochondral bone morphogenesis, osteoblast fate commitment, and vasculogenesis (Fig. S7d).

The Monocle^45^ method and RNA velocity analysis^46^ were used to identify chondrocyte differentiation trajectories. PreFCs were distributed along the trajectory and OssifyC at the end (Fig. 2e). RNA velocity analysis revealed the differentiation trajectories of PreFCs into FC-1s, FC-2s, and PathCs (Fig. 2f). In a previous study, OA patients with a significantly reduced PreFC have more severe chondrocyte damage and a significant increase in the percentage of FCs.^44^ And our differentiation trajectories results reveal the reasons for decreased PreFC and increased FCs.

We validated PreFC, FC-2, and OssifyC in OA cartilage using immunohistochemistry (IHC); SPP1(*OPN*) was used to identify OssifyC, *THBS1*, and *PIEZO2* were selected to identify PreFC, whereas PRG4(*Lubricin*) was selected to identify FC-2. We found that the *OPN*^+^ cells are significantly less at the superficial zone (SZ), and middle zone (MZ) in intact cartilage compared with damaged cartilage, and few *OPN*^+^ cells in the deep zone (DZ) of both groups (Fig. S9a). Meanwhile, the *THBS1*^+^ and *PIEZO2*^+^ cells are significantly more at the superficial zone (SZ), and middle zone (MZ) in intact cartilage than damaged cartilage, and there are few *THBS1*^+^ and *PIEZO2*^+^ cells in MZ of both groups (Fig. S9b, c). The *Lubricin*^+^ cells are significantly less at the superficial zone (SZ) in intact cartilage compared with damaged cartilage, and no *Lubricin*^+^ cells in the middle zone (MZ) and deep zone (DZ) of both groups (Fig. S9d). The IHC results confirm the proportion change of PreFC, FC-2, and OssifyC in intact and damaged cartilage.

Analysis of cellular communication patterns within cartilage and enriched 28 CCIs (Fig. S5b) revealed that PreFCs have high strength in receiving and sending CCIs in cartilage, and PreFCs transmit ANGPTL, MIF, FGF, GRN, PDGF, and BMP signals and receive VISFATIN, TGFb, MK, BMP, and GRN signals. ANGPTL,^47^ MK,^48^ and GRN^49^ signals promote cartilage repair, whereas VISFATIN signals lead to the degradation of the chondrocyte matrix.^50^ FGF,^51^ TGFb,^52^ and BMP^53^ signals regulate cartilage development and homeostasis. SPP1 and Postn signals are characteristic markers of OssifyC and PathC, respectively, and also mediate cartilage hypertrophy and ossification. These results indicate the pivotal role of PreFCs in maintaining cartilage homeostasis in the overall CCIs of cartilage.

### BM–cartilage crosstalk via CCI

**Fig. 3:**
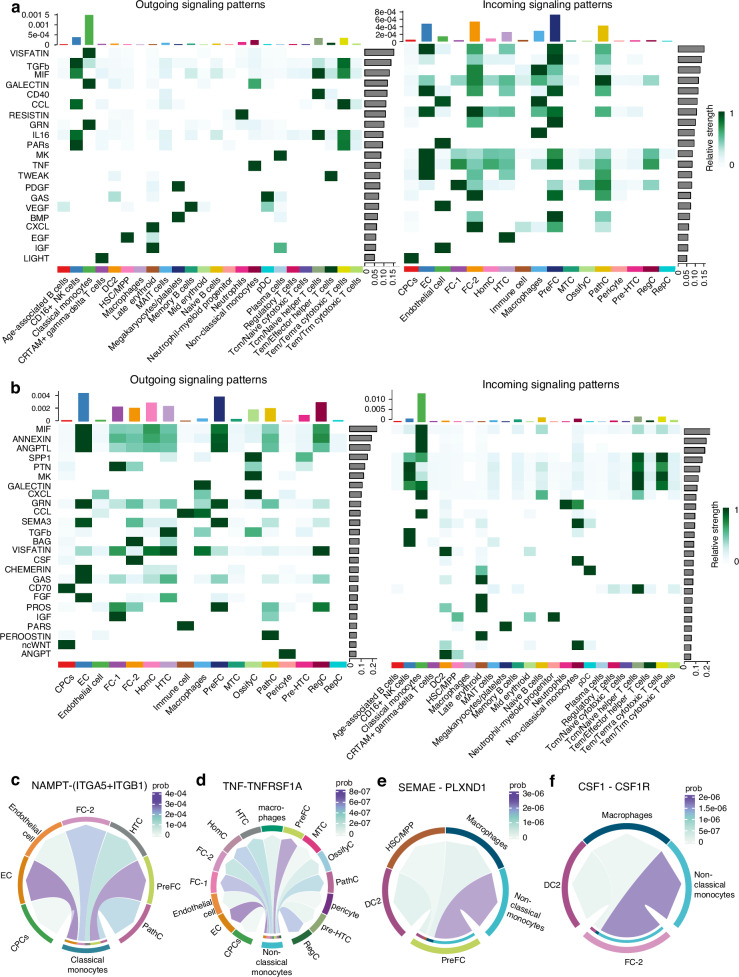
The CCIs between BM and cartilage clusters. **a** The relative strength of all enriched signals (outgoing and incoming) from cartilage to BM was visualized in a heatmap. **b** The relative strength of all enriched signals (outgoing and incoming) from BM to cartilage was visualized in a heatmap. **c** Chord diagram showing the ligand-receptor pair NAMPT–(ITGA5 + ITGB1) involved in CCIs between the classical monocytes and cartilage clusters. **d** The ligand-receptor pair TNF–TNFRSF1A is involved in CCIs between non-classical monocytes and cartilage clusters. **e** The ligand-receptor pair SEMA3E–PLXND1 is involved in PreFC and BM clusters. **f** The ligand-receptor pair CSF1–CSF1R is involved in FC-2 and BM clusters. CCI cell–cell interaction, NAMPT nicotinamide phosphoribosyltransferase, ITGA5 integrin subunit alpha 5, ITGB1 integrin subunit beta 1, TNF tumor necrosis factor, TNFRSF1A TNF receptor superfamily member 1A, SEMA3E semaphorin 3e, PLXND1 plexin d1, CSF1 colony stimulating factor 1, CSF1R colony stimulating factor 1 receptor, PreFC THBS1^+^ chondrocyte, BM bone marrow, FC-2 fibrocartilage chondrocyte -2

To understand the BM–cartilage crosstalk pattern, we used Cellchat^54^ to analyze the CCIs. The results indicated abundant CCIs between BM and cartilage, with 21 signals from BM to cartilage (Fig. 3a) and 25 signals from cartilage to BM (Fig. 3b). Pro-inflammation (MIF^55^) and cartilage homeostasis (GALECTIN,^56^ VISFATIN,^50^ and TGFb^57^) signals had high strength in BM-to-cartilage CCIs, suggesting that immune cells in the BM can promote an inflammatory response in cartilage and regulate cartilage homeostasis through the CCIs described above. Meanwhile, the pro-inflammatory (MIF,^36^ ANNEXIN,^35^ and ANGPTL^58^) signals have high strength in cartilage-to-BM CCIs, suggesting that cartilage modulates the inflammatory response of the BM.

Similar to the CCI patterns in cartilage, PreFCs are strong signal receivers and senders in cartilage among the BM-cartilage CCIs, indicating a pivotal role for PreFCs in cartilage and overall cartilage-BM cellular communication. Classical monocytes are the strongest signal receivers and senders in BM. They transmit the VISFATIN signal, which is related to cartilage degradation (Fig. 3a, c), and transmit the GRN signal, which suppresses the inflammatory action of TNF-α.^49^ Further, classical monocytes received chondrocyte-derived immune-related signals (MIF, GALECTIN, ANNEXIN, CXCL, and CCL) (Fig. 3b). MIF,^36^ GALCTIN,^59^ and ANNEXIN^35^ signaling can be pro-inflammatory, and CXCL, CCL, and other signals can recruit monocytes to the injured site and activate it. Non-classical monocytes are the only cluster that sends TNF signals to extensive chondrocyte clusters, and the EC and PreFC are the strongest receivers, suggesting that non-classical monocytes may promote inflammation and damage to chondrocytes through TNF signaling (Fig. 3d).

Bone remodeling of the subchondral bone is an essential feature of OA,^60^ and we found that non-classical monocytes might be the primary cell cluster for osteoclast formation in the subchondral bone; different cell clusters in cartilage regulate osteoclast formation. Specifically, non-classical monocytes received an osteoclast differentiation-inhibited SEMA3 signal from PreFCs (Fig. 3e) and the osteoclast formation CSF signal from FC-2 (Fig. 3f). Based on these results, non-classical monocytes may be the primary cell population for osteoclast formation in the subchondral bone, and osteoclast formation is regulated by chondrocytes in cartilage. The CCI results revealed the comprehensive BM–cartilage crosstalk, which plays a vital role in OA progression.

### The senescent non-classical monocyte is the signature of the immune landscape in BMLs

We confirmed the pro-inflammation role of non-classical monocytes in the progress of OA and BMLs. Meanwhile, the non-classical monocyte pro-inflammation CCIs between BMLs and cartilage resemble the senescence-associated secretory phenotype (SASP). Senescence is crucial in OA progression. Senescent cells influence other cells by secreting a senescence-associated secretory phenotype (SASP), leading to adverse effects. Therefore, applying the Gene Set Variation Analysis (GSVA) method, we used the Senmayo gene set,^61^ which has been validated in bone and BM scRNA-seq, to evaluate the senescence level in BM and cartilage samples from CONT and OA groups, as well as to assess the degree of senescence in each cell cluster.

**Fig. 4:**
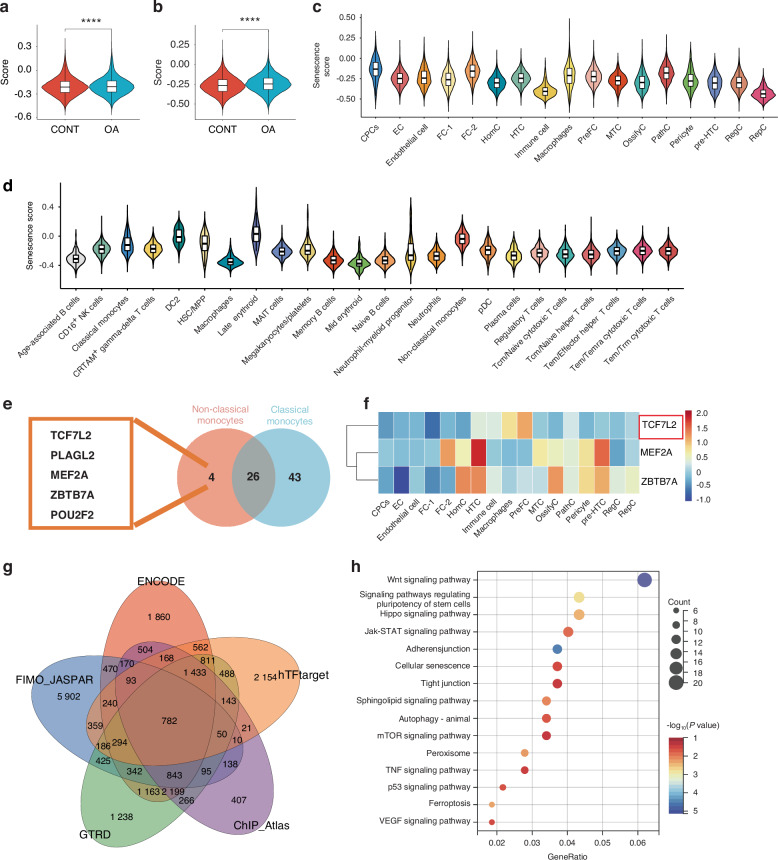
Assessment of the senescence degree of BM and cartilage and identifying the dysregulated transcription factors in the senescence of non-classical monocytes and chondrocytes. **a**, **b** Violin plots show higher senescence degrees of BMLs (**a**) and damaged cartilage(**b**). ****P < 0.000 1 by Wilcoxon test. **c**, **d** Violin plots showing the senescence degrees of each cluster in cartilage (**c**) and BM (**d**). **e** Five specific regulons of non-classical monocytes compared with classical monocytes. **f** The relative regulon activity of TCF7L2, MEF2A, and ZBTB7A in cartilage clusters are visualized in a heatmap. **g** Venn plots showing the predicted TCF7L2-target genes from ENCODE, FIMO, JASPAR, GTRD, hTFtarget, and ChIP_Atlas databases. **h** The dot plots show the KEGG enrichment results of the TCF7L2-target genes. BML, bone marrow lesion; FC-2, fibrocartilage chondrocyte-2

BM samples from BMLs (Fig. 4a), and damaged cartilage samples (Fig. 4b) had a significantly higher degree of senescence. Meanwhile, classical monocytes and non-classical monocytes have a high senescence score (Fig. 4d), and non-classical monocytes show a significantly higher level of senescence than classical monocytes (Fig. S10a). And non-classical monocyte (identified by CX3CR1), has a higher level of *p21* than the classical monocyte (identified by CCR2a), which was validated by immunofluorescence (IF) (Fig. S11a–c). The classical monocyte in the OA group has a significantly higher senescence score than in the CONT group (Fig. S10c). Besides, the majority of the chondrocyte clusters have higher senescence scores in damaged cartilage than intact cartilage (Fig. S10d).

We performed the pySCENIC^62^ analysis to understand the gene regulatory networks that differentiate the clusters and identify transcription factors specific to each cell cluster in BM and cartilage. We identified 294 enriched regulons in BM (Fig. S12, Table S6) and 346 in cartilage (Fig. S13, Table S7), and enriched regulons were highly specific to each cluster and regulons with high AUCell scores were plotted on the heatmap. Transcription factors with an AUCell score >0.5 were selected as the specific regulatory transcription factors of each cluster. Non-classical monocytes exhibit more hallmarks of senescence than classical monocytes and classical monocytes can transition into non-classical monocytes.^42^ Therefore, we used a Venn diagram to identify the specific transcription factors of non-classical monocytes, compared to classical monocytes. Five specific transcription factors that may regulate non-classical monocytes were identified—TCF7L2, PLAGL2, MEF2A, ZBTB7A, and POU2F2 (Fig. 4e).

Among the transcription factors mentioned above, only TCF7L2, MEF2A, and ZBTB7A participated in the gene regulation network of cartilage clusters, and TCF7L2 had high AUCell scores in PreFC and FC-2 (Fig. 4f), in which FC-2s are the chondrocyte clusters with the highest senescence level (Fig. 4c) in cartilage, and PreFCs can differentiate into FC-2s (Fig. 2g). FC-2 had a higher senescence score than PreFC (Fig. S10b), and FC-2 (identified by *Lubricin*), has a higher level of *p21* than classical monocyte (identified by *THBS1*) (Fig. S11d–f). Therefore, we speculated that the degree of senescence was upregulated during the transformation of PreFCs to FC-2s, and TCF7L2 is a senescence-related transcription factor that promotes cellular senescence of monocytes and chondrocytes.

We predicted the binding of TCF7L2 at target gene promoters in ENCODE, FIMO_JASPAR, GTRD, hTFtarget, and ChIP_Atlas databases by TF-Target Finder (TFTF),^63^ and applied the Venn diagram to intersect the target genes from each database. We obtained 782 possible TCF7L2-target genes (Fig. 4g). We performed an enrichment analysis of the above genes, and the results showed that the Wnt signaling pathway was the most dominant signaling pathway activated by TCF7L2, in addition to Cellular senescence, TNF signaling pathways, and p53 signaling pathways (Fig. 4h). The above results revealed that the activation of TCF7L2 led to cellular senescence and inflammation-related responses.

### TCF7L2 is a crucial regulator of senescence in chondrocytes and monocytes

**Fig. 5:**
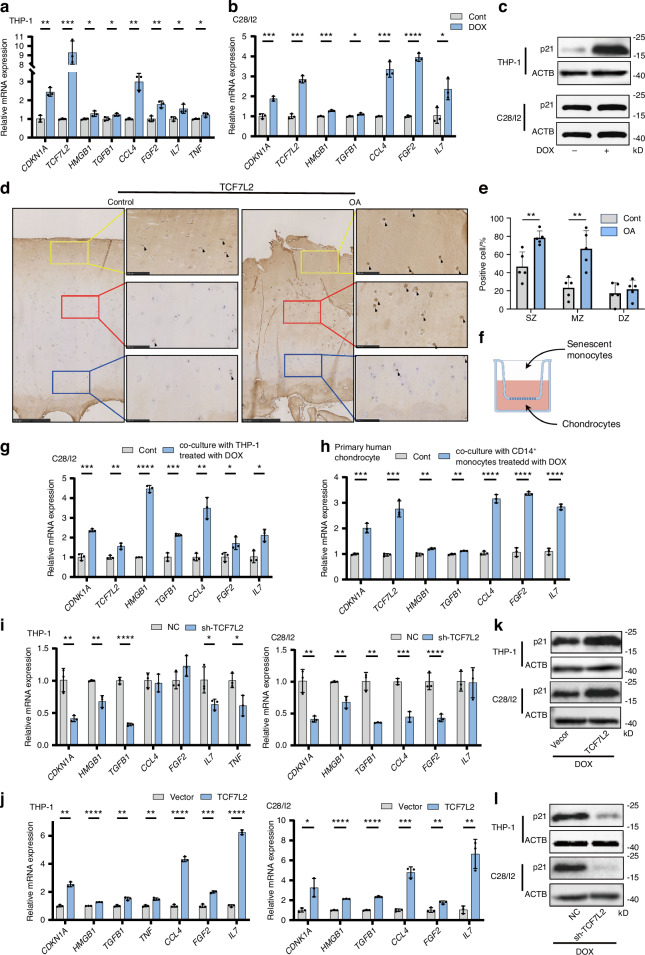
TCF7L2 is a crucial regulator of senescence in chondrocytes and monocytes. **a**, **b** DOX-treated THP-1 (**a**), C28/I2 (**b**) cells were harvested for RNA isolation and RT-qPCR analysis. **c** DOX-treated THP-1, C28/I2 cells were harvested for western blot analysis to detect the expression for P21. **d**, **e** The representative IHC images and quantification of TCF7L2 protein from different zones (ie, SZ, MZ, and DZ) in damaged and intact cartilage are displayed by bar plots (replicates *n* = 5). **f**–**h** The C28/I2 cells or human primary chondrocytes were co-cultured with DOX-treated THP-1or human primary CD14^+^ monocytes in Transwells for 4 days and harvested for RNA isolation and RT-qPCR analysis. **i** TCF7L2-activated THP-1, C28/I2 cells were harvested for RNA isolation and RT-qPCR analysis. **j** TCF7L2-depleted THP-1, C28/I2 cells using shRNA harvested for RNA isolation and RT-qPCR analysis. **k** TCF7L2-activated THP-1, C28/I2 cells were harvested for western blot analysis to detect the expression fo *P21*. **l** TCF7L2-depleted THP-1, C28/I2 cells were harvested for western blot analysis to detect the expression fo *P21*. Data are relative to β-actin and normalized to control, vector, or NC shRNA. BML bone marrow lesion, IHC immunohistochemistry, IF immunofluorescence. DOX doxorubicin, *P < 0.05, **P < 0.01, ***P < 0.001, ****P < 0.000 1 by multiple unpaired Student’s *t*-test

Certain chemotherapeutic drugs such as doxorubicin (DOX) are potential inducers of senescence, and DOX stimulation is an established method for investigating senescence in monocytes^64^ and chondrocytes^65^ in vitro. We used 100 nmol/L DOX to treat human chondrocytes and C28/I2 cells for 4 days, and 15 nmol/L DOX to induce senescence in human monocytes (THP-1) for 7 days. Subsequently, we tested CDKN1A and *p21* expression to confirm the senescent status of the cells as well as the expression of TCF7L2 and SASP-related genes. Senescent chondrocytes and monocytes had higher expression levels of TCF7L2, CDKN1A, *p21* and SASP profiles, including HMGB1, TGFB1, CCL4, FGF2, and IL7. TNF expression was upregulated during DOX-induced senescence (Fig. 5a–c). The enhancement of TCF7L2 in damaged cartilage was validated using immunohistochemistry (IHC), and *TCF7L2*^+^ cells are significantly enriched in the SZ and MZ of damaged cartilage (Fig. 5d, e), and immunofluorescence (IF) results showed that *TCF7L2* was highly expressed in damaged cartilage, but not in the intact area (Fig. S14).

A Transwell assay was conducted to evaluate the impact of senescent monocytes on chondrocytes. Senescent monocytes (THP-1 cells) were seeded in the cell culture insert and co-cultured with C28/I2 cells for four days. CDKN1A, TCF7L2, and SASP genes, including HMGB1, TGFB1, CCL4, FGF2, IL7, were upregulated in C28/I2 cells (Fig. 5f, g). Meanwhile, we isolated the human primary chondrocytes and CD14^+^ monocytes from human articular. The CD14^+^ monocytes was treated with DOX to induce the senescence, and co-cultured with human primary chondrocyte for four days. CDKN1A, TCF7L2, and SASP genes, including HMGB1, TGFB1, CCL4, FGF2, IL7, were upregulated in human primary chondrocyte human primary chondrocytes (Fig. 5h).

Further, C28/I2 and THP-1 cells were transfected with a TCF7L2 overexpression plasmid, and CDKN1A and SASP genes (HMGB1, TGFB1, CCL4, FGF2, IL7) were upregulated in both cell types and TNF in monocytes (Fig. 5i). Furthermore, we induced senescence in C28/I2 and THP-1 cells, and used TCF7L2 shRNA to deplete TCF7L2, and significantly reduced the expression of CDNK1A and the SASP genes, including HMGB1, TGFB1, and IL7 (Fig. 5j), and depletion of TCF7L2 downregulated TNF expression of THP-1 cells. Furthermore, the overexpression of TCF7L2 increased the level of *p21* in c28/i2 and THP-1 cells (Fig. 5k). The depletion of TCF7L2 decreased the expression of *p21*, which is induced by the DOX in c28/i2 and THP-1 cells (Fig. 5l). These data suggest that TCF7L2 is a crucial regulator of cell senescence in chondrocytes and monocytes and induces CDKN1A and SASP gene expression.

## Discussion

**Fig. 6:**
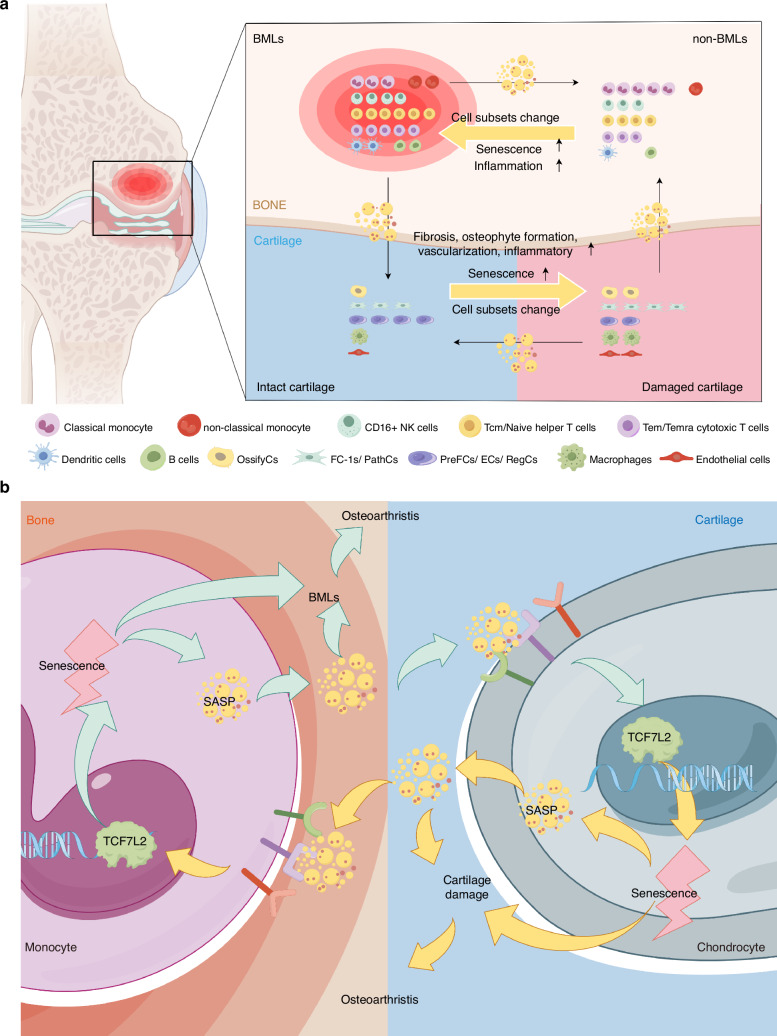
BMLs promoting OA progression. **a** BMLs and damaged cartilage change the cell subset composition of non-BMLs and intact cartilage, promote the senescence and inflammation of non-BMLs, and senescence, fibrosis, osteophyte formation, vascularization, and inflammation of intact cartilage. **b** TCF7L2 promotes the senescence of monocytes and chondrocytes, inducing BMLs and cartilage damage, respectively, which promotes OA progression. BML bone marrow lesion. TCF7L2, transcription factor 7 like 2; OA, osteoarthritis. The figures were drawn by Figdraw

In previous studies, Zeng resolved the cell clusters in BMLs from bulk-RNA data by back-convolution,^66^ whereas Hu analyzed the subchondral bone cell populations without focusing on BMLs, and the study lacked a control group. Therefore, the above studies may not reveal the complete immunologic landscape of BMLs. Also, none of the above studies discussed the role of BMLs on cartilage damage in OA progression. We performed extensive integrative analysis of non-BML and BML tissues in the BML region of patients with OA and cartilage in the intact and damaged cartilage regions and revealed a pro-inflammatory immune landscape of BMLs in which non-classical monocyte is the main subset promoting OA. The Pre-FC can maintain cartilage homeostasis and is exhausted in damaged cartilage. We reveal the crosstalk between BMLs and cartilage and how BMLs and damaged cartilage promote the progression of each other, as shown in Fig. 6a. Our study demonstrates an elevated level of senescence in BMLs and damaged cartilage, and the non-classical monocyte is the most senescent subset in BMLs, which promotes the senescence of chondrocytes. We identify that TCF7L2 promotes senescence and regulates the senescence-associated secretory phenotype (SASP) of monocytes and chondrocytes, a candidate therapeutic target in OA, as shown in Fig. 6b.

Our results reveal the BML immune landscapes. Although the proportion of the cell clusters has no significant change between non-BML and BML, the significantly higher inflammation score and OA score, and the enrichment of up-regulated genes in BMLs reveal a stronger pro-inflammation and pro-OA effect of classical and non-classical monocytes in BMLs. Therefore, the biological process changes of clusters indicated that the BM tissue in BMLs had a more pro-inflammatory immune landscape. During OA initiation, non-classical and classical monocytes are recruited to damaged cartilage and activated.^38,67,68^ Classical monocytes can transform into non-classical monocytes to help sustain inflammation in the later stages of OA.^38^ We found that classical monocytes receive CCL and CXCL signals of cartilage origin that promote monocyte migration and pro-inflammatory Galectin and MIF signals.

Recent studies have demonstrated that PreFC can act as a preventative cluster against cartilage degeneration,^44^ and OA patients exhibiting a reduced percentage of PreFC demonstrated a greater degree of cartilage damage, accompanied by a significant escalation in the proportion of FC and the activation of inflammation-related pathways.^44^ In our study, we found that PreFC is involved in multi-biological processes involved in cartilage homeostasis. In damaged cartilage, the proportion of PreFC was significantly reduced, and concomitantly, PreFC was able to differentiate into FC-1, FC-2, and PathC, which may be the reason for the PreFC reduction and increase in the proportion of FC. Therefore, the inhibition of the differentiation of PreFC was able to inhibit the OA in the levels of cartilage damage. Meanwhile, PreFCs are crucial for sending and receiving signals within the cartilage and between the cartilage and BM. These cells transmit numerous CCIs to maintain cartilage homeostasis but are depleted by detrimental CCIs originating from BMLs and other subsets. This depletion was evidenced by a significant decrease in PreFCs in the damaged cartilage.

Non-classical monocytes are the only senders of pro-inflammatory TNF signals to chondrocytes. Simultaneously, classical monocytes transmit the GRN signal that protects the cartilage, which can inhibit the pro-inflammatory effect of the TNF signal,^49^ indicating that there is a balance between classical monocytes and non-classical monocytes in BM–cartilage crosstalk, which is broken in BMLs, leading to damage in the cartilage. PreFC is one of the primary receivers of TNF and GRN signals, indicating that PreFC is the key chondrocyte cluster in BM–cartilage CCIs. A study showed that TNF-α resulted in increased expression of SPP1, and PRG4 of chondrocyte,^69^ which are the markers of OssifyC and FC-2, respectively. Concurrently, TNF-α treatment elevated the expression of several chemokines of chondrocytes, including CXCL, CCL, and CFS signal,^69^ which have been demonstrated to play pivotal roles in the regulation of inflammation and the recruitment of immune cells. Furthermore, we found that CCIs from the cartilage regulate the osteoclast differentiation of non-classical monocytes, which have a higher potential for osteoclast differentiation,^70^ and our results indicate that non-classical monocytes may be the primary cell population for osteoclast formation in the subchondral bone. We also found many CCIs in BM and cartilage. The above results provide insights into the role of the close crosstalk between BMLs and joint cartilage in OA progression from the CCI perspective.

Cellular senescence, a hallmark of aging,^71^ can be triggered by exogenous stressors^72^ and is characterized by the release of deleterious pro-inflammatory molecules into the surrounding microenvironment, known as the SASP, ultimately forming the distinct senescence-inflamed niche.^73,74^ Recent increasing studies highlighted the critical role of subchondral bone cell senescence in mediating subchondral architectural alterations and OA progression.^75^ Cellular senescence via SASP promotes the accumulation of senescent phenotypes and elevated production of pro-inflammatory factors of multiple cells in subchondral bone, including mesenchymal stem cells,^76^ osteoclast,^77^ and immune cells.^75^ Senescence-associated immune profiles in BMLs provide a potential mechanistic link between immune responses to injury, senescence, and aging.^11^ We confirmed the presence of a senescent immune landscape in BMLs and a higher level of cellular senescence in damaged cartilage. We also demonstrated a high degree of senescence in non-classical monocytes. Given the critical role of bone–cartilage crosstalk, increased senescence in BMLs also contributes to OA. Further, the pro-inflammatory phenotype of non-classical monocytes is attributed to senescence,^42^ and we demonstrated that senescent monocytes have a stronger SASP profile and can induce senescence in chondrocytes.

We also identified that TCF7L2, a crucial transcription factor in the WNT signaling pathway, regulates the senescence of monocytes and chondrocytes (Fig. 6b). TCF7L2 is essential to osteoarthritis progression, and TCF7L2 overexpression induces the expression of OA-related catabolic enzymes and ADAMTS4 in human chondrocytes.^78^ In addition, it has also been suggested that TCF7L2 is involved in mechanical signaling in chondrocytes during the development of osteoarthritis disease, leading to chondrocyte atrophy.^79^ And TCF7L2 is an important transcription factor that regulates the differentiation of monocytes into macrophages.^80^ And, we predicted the bind of TCF7L2 in the promoter of target genes, and the WNT signaling pathway, Cellular senescence, TNF signaling pathways, and p53 signaling pathways are enriched. Moreover, increased TCF7L2 expression was observed in senescence-induced monocytes and chondrocytes. TCF7L2 overexpression in monocytes and chondrocytes enhanced the expression of core senescent CDKN1A (*p21*) and increased the expression of SASP-related genes, suggesting a direct role for TCF7L2 in promoting senescence. The inhibition of TCF7L2 expression in monocytes and chondrocytes after senescence induction reduced the expression of CDKN1A (*p21*) and partially inhibited the SASP-related gene expression. Therefore, TCF7L2 is a potential therapeutic target for the treatment of OA.

This study could be improved in several ways. There has been significant progress in spatial single-cell transcriptome sequencing.^44^ Senescent cells can affect neighboring cells by secreting SASP, making spatial single-cell transcriptome research uniquely advantageous for investigating senescent cells and SASP-related research. Here, the Senmayo dataset was used to determine the senescence status of individual cells in OA cartilage and BM samples. We propose that employing SPiDER-β-Gal fluorescent probe and flow cytometry sorting techniques may enable the segregation of senescent and non-senescent cells for subsequent joint analysis following scRNA sequencing, presenting a viable approach.

Despite these limitations, our study presents a transcriptional landscape of bone marrow in non-BML/BML, and intact/damaged knee articular cartilage at single-cell resolutions. Furthermore, our study reveals the crosstalk between the bone marrow and cartilage. We found that senescent non-classical monocytes promote BMLs and inflammation and senescence of chondrocytes by modulating BM–cartilage crosstalk in OA, with TCF7L2 serving as a regulator. The new findings have the potential to enhance diagnosis and treatment, provide insights into key molecular players in OA, and uncover promising drugs for personalized medicine strategies in OA.

## Materials and methods

### Human knee tissue collection

All human tissues were obtained with approval by the Ethics Committee of the First Affiliated Hospital of Zhejiang University of Chinese Medicine. The bone marrow samples of non-BML and the BML areas were obtained from 4 donors who accepted unicompartmental knee replacement (3 females and 1 male; age 67-80; mean ± SE 72.5 ± 6.31). MR images were obtained with a 3.0 T MRI system (GE) MRI was performed using fat-suppressed, fast spin-echo, proton density-weighted sequences. T1-weighted sequences were obtained in 1 plane to identify BMLs. To ensure accurate cutting measurements in 3 dimensions for separation of BML from non-BML regions, MRI sequences were obtained in 3 planes (axial, coronal, and sagittal). BMLs were defined as areas of increased signal intensity adjacent to the subcortical bone on proton density-weighted sequences that had low intensity on T1-weighted sequences. Identification of BML and non-BML regions was repeated independently by a radiologist with expertise in musculoskeletal imaging. Consensus regarding BML and non-BML regions was then reached by discussion. Because BMLs have an ill-defined border, a 2-3 cm gap was left between BML and non-BML regions to ensure that BML was well separated from non-BM and used bone marrow puncture needles to extract the bone marrow samples from non-BML and BML areas during the operation.^81^ The matching cartilage samples were obtained from the same knee. Full-thickness cartilage samples were harvested from the intact cartilage overlay of the non-BMLs and the damaged cartilage overlay of the BMLs, respectively. Details of donors are included in Table S8.

### Articular cartilage and bone marrow single-cell isolation

Human cartilage tissue was washed with phosphate-buffered saline (PBS) and cut into 0.5 mm^3^ pieces. Samples were then pretreated with 0.25% trypsin at 37 °C in an incubator with 5% CO_2_ for 30 min. The cartilage fragment was centrifuged at 350 × *g* for 5 min, and the supernatant was discarded completely and dissociated into single cells in 0.2% collagenase II (diluted in basal Dulbecco’s Modified Eagle) for 10 h at 37 °C. The resulting cell suspension was filtered by passing through a 70 μm cell strainer and centrifuged at 300 × *g* for 5 min at 4 °C to collect the cell pellet. The human bone marrow tissue was filtered by passing through a 70 μm cell strainer and centrifuged at 300 × *g* for 5 min at 4 °C to collect the cell pellet. After lysing erythrocytes (MACS 130-094-183, 10×), Dead Cell Removal MicroBeads (MACS 130-090-101, 10×) and Miltenyi Dead Cell Removal Kit (MACS 130-090-101, 10×) were used to remove dead cells. Then, the resulting cell suspension was filtered by passing through a 70 μm cell strainer in PBS (0.04% BSA) and washed twice with PBS buffer. The cell pellet was resuspended in 50 μL PBS (0.04% BSA). The overall cell viability was confirmed by trypan blue exclusion, which needed to be above 85%. Single-cell suspensions were counted using a hemocytometer/Countess II Automated Cell Counter, and concentration was adjusted to 700–1 200 cells/μL.

### Library preparation and single-cell RNA sequencing

Single-cell suspensions were loaded to 10× Chromium to capture 10 000 single cells according to the manufacturer’s instructions for 10× Genomics Chromium Single-Cell 3’kit (V3). The following cDNA amplification and library construction steps were performed according to the standard protocol. Libraries were sequenced on an Illumina NovaSeq 6000 sequencing system (paired-end multiplexing run,150 bp) by LC-Bio Technology co.ltd., (Hangzhou, China) at a minimum depth of 20 000 reads per cell.

### scRNA-seq data processing

Sequencing results were demultiplexed and converted to FASTQ format using Illumina bcl2fastq software (version 2.20). Cell Ranger counts were used to align the reads to the human reference genome (GRCh38) and to generate count matrices for each gene for each cell. Filtered feature barcode matrices generated from Cell Ranger were exported into Seurat (v4.1.0) to generate individual Seurat objects. Seurat workflows were used for quality control, sample integration, downscaling, and clustering of individual datasets.^32,82,83^ The quality control threshold was as follows: all genes expressed in less than three cells were removed, 500 feature minimum counts and 5 000 feature maximum counts were limited for each object, respectively, UMI counts less than 500, the percent of mitochondrial-DNA derived gene expression <25%.

### Dimensionality reduction and clustering

To visualize the data, we further reduced the dimensionality of all cells using Seurat and used UMAP (Uniform Manifold Approximation and Projection) to project the cells into 2D space. We use the LogNormalize method of the Seurat software to calculate the expression value of genes. Then, PCA (Principal component analysis) analysis was performed using the normalized expression value; within all the PCs, the top 20/34 PCs for cartilage/bone marrow were used to do clustering and UMAP analysis, which was determined as the optimal number of components for dimensionality reduction by an elbow plot. Clustering was performed with the FindClusters functions in Seurat, using a resolution of 0.7 for the cartilage sample and 0.8 for the bone marrow sample.

### Annotating cell clusters

Marker genes for each cluster were identified using the “bimod” method with default parameters via the FindAllMarkers function in Seurat. These selected marker genes are expressed in more than 10% of the cells in a cluster, the average log (Fold Change) is more significant than 0.26, and the *P*-value is less than 0.01. The Cluster-specific differentially expressed genes (DEGs) were used to enrich Gene Ontology (GO) terms by clusterProfiler^84^ packages (version 3.14.3). For clusters in cartilage samples, the previously reported cellular biomarkers and GO were utilized to annotate the cell clusters. For clusters in bone marrow samples, CellTypist^30^ (https://www.celltypist.org/, immune V2), a pan-tissue immune database and a tool for automated cell type annotation, was used to auto annotate cell clusters, with the parameter low-hierarchy (high-resolution, 91 cell types and subtypes) classifier. Then, manually calibrate the annotation results with previously reported cellular biomarkers and GO.

### Cell-cell interactions analysis (Cellchat)

Cell-cell interaction analyses were performed using CellChat^54^ (V.1.4.0). CellChat was run on each group, and the log-normalized expression data and cell annotation information were analyzed. We followed the standard pipeline to perform all analyses using the default parameters and the human ligand-receptors database.

### Pseudotime analysis (Monocle2)

Pseudotime is a progression of cells along a virtually estimated path, mimicking temporal development. Using Monocle, an independent component analysis (ICA) dimensional reduction is performed, followed by a projection of a minimal spanning tree (MST) of the cell’s location in this reduced space. Each cell is assigned a pseudotemporal space (100,101). Monocle 2^45^ was used to preprocess, perform UMAP reduction, and reduce the dimensionality using the DDRTree algorithm with a maximum of four dimensions. Subsequently, the cells were ordered, and genes were plotted along the reduced dimension. Differential gene testing has been performed with the formula “~sm. ns(Pseudotime)”, and the results were restricted by a *q* value < 0.1(100).

### RNA velocity

Velocyto.^85^ (v.17.17) was run on the Cellranger counts output folder to generate a loom file containing the spliced/unspliced counts matrix. The loom file was merged to an annadata object containing cartilage and bone marrow counts matrix metadata. The spliced/unspliced counts were then normalized using the package scVelo.^46^ (v0.2.5), and genes without a shared splice count of 20 were filtered out of the annadata object. Velocities were estimated using scVelo’s dynamical model, and velocities were then calculated and embedded onto UMAPs.

### Gene regulatory network analysis (pySCENIC)

The Python implementation of Single-Cell Regulatory Network Inference and Clustering (pySCENIC).^62^ was used to identify gene regulatory networks (GRNs) that regulate cartilage and bone marrow cell types. Genes were first soft-filtered to remove background noise. These expression matrices were subjected to GRN analysis using GRNBoost2 to identify transcription factors (TFs) and target genes with significant co-expression patterns. RcisTarget analysis used the Motif database (hg19-500 bp upstream and hg19-tss-centered-10 kb), creating modules in which binding Motifs that regulate TFs are significantly enriched in target genes. The resulting “regulatory factors” include regulatory transcription factors and all their direct gene targets. Finally, the Regulatory Factor Activity Score for each cell was calculated using AUCell and integrated into the metadata of the Seurat object. The Regulatory Factor Scores for the Single Cell Transcription Factor Module for individual cells are integrated into the metadata of the Seurat object and then displayed as heatmaps or UMAP plots.

### Inflammation, OA, and senescence processes scoring

An inflammation gene set,^86^ and an OA gene set.^24^ (Table S3) were used to assess the contribution to the OA progression and pro-inflammation of each cell cluster by the AddModuleSocre module of Seraut. And the SenMayo gene set was used to assess the senescence level of each cluster.^61^

### Animals

C57BL/6 J mice for modeling were obtained from Zhejiang Chinese Medical University Laboratory Animal Research Center. All mice were housed at 21 °C ± 2 °C with a relative humidity of 40% ± 5%. The room was maintained on a 12-h light/dark cycle with free access to water and lab chow. All studies were approved by the Committee on the Ethics of Animal Experiments of Zhejiang Chinese Medical University.

### DMM OA mouse model

DMM (destabilization of the medial meniscus) surgery was performed on the right knee joints of mice, as previously described.^87^ Briefly, male C57BL/6 mice were performed DMM with anesthetized, and a 3 mm longitudinal incision was made on the medial part of the left knee. The medial meniscotibial ligament of the left joint was exposed and was transected to give destabilization of the medial meniscus. Then, the medial joint capsule was sutured, and the skin was closed.

### Immunohistochemistry (IHC) and immunofluorescence (IF)

The human cartilage tissue was decalcified with 10% EDTA solution. Human cartilage samples were embedded in paraffin blocks and cut into 5 µm sections. Antigen retrieval was performed with sodium citrate buffer at 70 °C for 30 min. The deparaffinized and hydrated histological slides of human cartilage tissue were blocked by 5% bovine serum albumin (BSA) for 1 h at room temperature and incubated overnight (4 °C) with primary antibodies against *TCF7L2*, *THBS1*, *PIZEO2* (13838-1-AP, 18304-1-AP, 26205-1-AP, Proteintech, Wuhan, China), *OPN* (ab8448, Abcam, UK), and *Lubricin* (ER1912-53, HUABIO, China). After staining with 3,3’-diaminobenzidine and counterstaining with hematoxylin, tissue sections were dehydrated in gradient ethanol and sealed with a neutral balsam mounting medium. Typical positive cells were captured under a microscope.

For immunohistochemical staining, 3% (v/v) H_2_O_2_ was used to quench the endogenous peroxidase activity, and primary antibodies against *TCF7L2*, *THBS1, CCR2a, CX3CR1* (13838-1-AP, 18304-1-AP, 16153-1-AP, 13885-1-AP, Proteintech, Wuhan, China), and *Lubricin* (ER1912-53, Huabio, Hangzhou, China) were incubated overnight at 4 °C. After washing, the sections were incubated with Alexa Fluor 555 Antibody Labeling Kits (A20187, ThermoFisher Scientific), CoraLite488-conjugated goat anti-rabbit IgG and CoraLite594-conjugated goat anti-mouse IgG (SA00013-2, SA00013-3, Proteintech, Wuhan, China). DAPI was counterstained and observed under a fluorescence microscope.

### Cell culture and senescence induction

The human chondrocyte cells, C28/I2 cells, were cultured in DMEM with 10% (v/v) calf serum (CS) supplemented with 1% (v/v) penicillin-streptomycin-glutamine (PSG) and streptomycin at 37 °C and 5% CO_2_ condition. The human monocyte cell, THP-1, were cultured in RPMI-1640 medium containing 10% (v/v) calf serum (CS) supplemented with 1% (v/v) penicillin-streptomycin-glutamine (PSG) at 37 °C and 5% CO_2_ condition. The primary chondrocytes were isolated from the articular cartilage tissues from the doners accepted total knee arthroplasty. The isolated process is as described in “Articular cartilage and bone marrow single-cell isolation” section. And the primary chondrocytes were cultured in DMEM with 10% (v/v) calf serum (CS) supplemented with 1% (v/v) penicillin-streptomycin-glutamine (PSG) and streptomycin at 37 °C and 5% CO_2_ condition. The primary human CD14^+^ monocytes was isolated by the human CD14^+^ cell isolation kit (positive selection) (91-01-0012, Xinbio, Suzhou, China) from the human bone marrow, and were cultured in RPMI-1640 medium containing 10% (v/v) calf serum (CS) supplemented with 1% (v/v) penicillin-streptomycin-glutamine (PSG) at 37 °C and 5% CO_2_ condition.The cell culture medium was replenished every 72 h. C28/I2 cell was treated with 100 μmol/L doxorubicin.^88^ (DOX, S17092, Yuanye, Shanghai, China) for 4 days, and THP-1 cell and primary human CD14^+^ monocyte was treated with 15 μmol/L doxorubicin.^64^ for 7 days to induce senescence.

### Transwell assay

C28/I2 cells or primary chondrocytes were grown to confluency in 24-well plates and then co-cultured in a transwell system with senescence THP-1 or primary CD14^+^ monocytes, respectively. THP-1 cell and primary CD14^+^ monocytes were treated with 15 μmol/L doxorubicin (DOX, S17092, Yuanye, Shanghai, China) for 7 days to induce senescence. After subsequent centrifugation to remove the supernatant, the cells were washed with PBS and centrifuged again, followed by resuspension of the cells and a total cell number of 1 × 10^5^ to the upper chamber of the transwell. C28/I2 cells were co-cultured with THP-1 cells, and primary chondrocytes were co-cultured with primary CD14^+^ monocytes, with no DOX treated for controls. Co-culture experiments were carried out for 72 h.

### Reverse transcription-quantitative polymerase chain reaction (RT-qPCR)

According to the manufacturer’s instructions, RNA was extracted from cells using the SPARKeasy Cell RNA Kit (AC0205, SparkJade, Shandong, China). Reverse transcription used the EVO M-MLVRT Mix kit (AG11728, Accurate Biology, China) by the standard protocol. qPCR assays were performed using SYBR® Green Premix Pro Tag HS qPCR Kit (AG11718, Accurate Biology, China) and primers listed in Table S9. For each gene, the relative mRNA expression level was normalized to the expression level of β-actin and calculated using the 2^-∆∆CT^ method.

### Western blot analysis

Total proteins were collected immediately in a lysis buffer containing protease and phosphatase inhibitors, incubated on ice for 30 min, and separated on a 12% SDS PAGE gel. The samples were then transferred onto polyvinylidene fluoride membranes and blocked in 5% bovine serum albumin for 1 h. Immunoblotting was subsequently performed by incubating the membranes overnight at 4 °C with rabbit primary antibodies targeting *p21* (A19094, Abclone, Wuhan, China). The membranes were incubated with goat anti-rabbit horseradish peroxidase-conjugated secondary antibody (SA00001-0, Proteintech, Wuhan, China) at room temperature for 1 h. The densities of the bands were determined and normalized to Actb.

### cDNA overexpression experiments

C28/I2 cells were transfected with overexpression vectors containing cDNA for TCF7L2 using GP-transfect-Mate (GenePharma, Suzhou, China). RNA was extracted 24 h post-transfection using the above protocols, and RT-qPCR assays were performed. Data were normalized individually to the empty vector control.

### shRNA depletion experiments

C28/I2 cells were transfected with shRNAs targeting TCF7L2 (5’-CAAGATGGAGGGCTCTTTAAG- 3’) using GP-transfect-Mate (GenePharma, Suzhou, China). RNA was extracted 24 h post-transfection using the above protocols, and RT-qPCR assays were performed. Data were normalized individually to a non-targeting control (NC) shRNA.

### Statistical analysis

All quantitative data are expressed as means ± SE (standard error) or SD (standard deviation). Results were analyzed using GraphPad Prism version 9.5 (GraphPad Software, San Diego, CA). A two-tailed unpaired Student’s *t*-test for parametric data was used to establish statistical significance between the two groups. Statistical tests used are described in each figure legend. *P*-values below 0.05 were considered significant. No multiple comparisons were performed.

## Supplementary information

Repetitions of uncropped gels for Westem Blots 1

Repetitions of uncropped gels for Westem Blots 2

Supplement materials

Supplementary Table 1

Supplementary Table 2

Supplementary Table 3

Supplementary Table 4

Supplementary Table 5

Supplementary Table 6

Supplementary Table 7

Supplementary Table 8

Supplementary Table 9

## Data Availability

Data will be available in GEO.
