# Supplementary material for: Single-cell sequencing reveals a senescent immune landscape in bone marrow lesions inducing articular cartilage damage in osteoarthritis
Source: Bone Res. 2025 Nov 21;13:94. doi: 10.1038/s41413-025-00467-4 (PMC12639131; doi:10.1038/s41413-025-00467-4)
Supplement: Supplementary file 3 — Supplement materials [file 41413_2025_467_MOESM3_ESM.docx]

**Supplement materials**



**SUPPLEMENTARY FIGURES:**

Supplementary Figure 1. Interrogation of non-BMLs and BML clusters. (A, B) UMAPs show the contribution of cells from each condition (A) and each donor (B) to the integrated Seurat object. (C) The percentage of total cells in the integrated dataset originates from non-BMLs and BML donors. (D) UMAPs show the contribution of each cell cluster in non-BML (CONT, left) and BML (OA, right). (E) Percentage of cells contributing to each cluster in non-BML and BML. (F)  The quantification of each subset in CONT and OA is shown as a cell count percentage.



 Supplementary Figure 2. The dot plot shows the mean expressions of the marker genes for cell subsets.



 Supplementary Figure 3. The Violin plots show the statistical analysis of the inflammatory (A) and OA (B) scores of each cluster of bone marrow in the CONT and OA group. * *P*<0.05, ** *P*<0.01, *** *P*<0.001, **** *P*<0.0001 by Wilcoxon test.





Supplementary Figure 4. Dot plot showing the GO and KEGG enrichment results of the up-regulated genes of classical monocytes (A) and non-classical monocytes (B) in the OA group compared with the CONT group.



Supplementary Figure 5. The relative strength of all enriched signals (outgoing and incoming) across BM (A) and cartilage (B) clusters as visualized in heat maps.



 Supplementary Figure 6. Interrogation of intact and damaged cartilage clusters. (A, B) UMAPs show the contribution of cells from each condition (A) and each donor (B) to the integrated Seurat object. (C) The percentage of total cells in the integrated dataset originates from intact and damaged cartilage donors. (D) UMAPs show the contribution of each cell cluster in intact cartilage (CONT, left) and damaged cartilage (OA, right). (E) Percentage of cells contributing to each cluster in intact cartilage and damaged cartilage. (F) The quantification of each subset in CONT and OA is shown as a cell count percentage. The quantification of each subset in CONT and OA is shown as a cell count percentage.



Supplementary Figure 7. The gene markers and biological process of PreFCs and ossify chondrocyte (OssifyC) (A)Violin plots showing expression of top 15 gene markers of PreFCs in each cluster. (B) Dot plot showing the top 20 biological processes of the PreFCs. (C)Violin plots showing expression of top 15 gene markers of ossify chondrocyte (OssifyC) in each cluster. (D) Dot plot showing the top 20 biological processes of the OssifyC.



 Supplementary Figure 8. Dot plot showing the KEGG (A) and GO (B) enrichment results of the up-regulated genes of PreFCs in the OA group compared with the CONT group.



Supplementary Figure 9. (A-D) Representative immunohistochemistry assay of indicated genes of OssifyC (OPN) (A), PreFC (*THBS1*(B), *PIZEO2*(C)) and FC-2 (*Lubricin*) (D)in damaged and intact cartilage. The quantification of positive cells from different zones (ie, SZ, MZ, and DZ) is displayed by bar plots (replicates n=5) in cartilage. OssifyC, ossify chondrocyte; PreFC, prefibro chondrocyte; FC, fibrocartilage chondrocytes. SZ; superficial zone; MZ, middle zone; DZ, deep zone. *P<0.05, ** P<0.01, **** P<0.0001 by unpaired Student’s t-test.



Supplementary Figure 10. The Violin plots show that non-classical monocytes have higher senescence scores than classical monocytes (A), and the FC-2s have higher senescence scores than PreFC (B). The Violin plots show the statistical analysis of the senescence scores of each cluster of bone marrow (C) and cartilage (D) in the CONT and OA groups. *P* values , * *P*<0.05, ** *P*<0.01, *** *P*<0.001, **** *P*<0.0001 by Wilcoxon test.



 Supplementary Figure 11. The representative IF images of *p21* (red) and marker of (A) classical monocytes (*CCR2*, green), (B) non-classical monocytes (*CXCR1*, green), (C) PreFC (*THBS1*, green), and (D) FC-2 (*Lubricin*, green) in DMM-induced OA mice articular. The comparison of quantified fluorescence intensity of *p21* between *THBS1*^high^ and *Lubricin*^high^ cells (C), as well as CCR2^high^ and CXCR1^high^ cells (F). *P* value, ** *P*<0.01, *** *P*<0.00 by unpaired, 2-tailed Student’s t-test.



Supplementary Figure 12. pySCENIC analysis revealed enriched regulons across all bone marrow clusters. (A) Relative regulon activity (AUCell score) of transcription factors of each cell is visualized in a heatmap. (B, C) pySCENIC analysis revealed enriched regulons of classical monocytes (B) and non-classical monocytes (C). The relative regulon activity (AUCell score >0.5 a) of transcription factors is visualized in a heatmap.



 Supplementary Figure 13. pySCENIC analysis revealed enriched regulons across all articular cartilage clusters. Relative regulon activity (AUCell score) of transcription factors of each cell is visualized in a heatmap. (B, C) pySCENIC analysis revealed enriched regulons of Pre-FC (B) and FC-2 (C). The relative regulon activity (AUCell score >0.5 a) of transcription factors is visualized in a heatmap.





Supplementary Figure 14. The representative IF images of TCF7L2 protein in damaged and intact articular cartilage.





Supplementary Figure 15. Uncropped gels for Westem Blots in Figure 5
